# Functional organization and regulatory logic of the *ped* gene cluster in *Pseudomonas* species

**DOI:** 10.1128/aem.00046-26

**Published:** 2026-02-18

**Authors:** Òscar Puiggené, Pablo I. Nikel

**Affiliations:** 1 BRiGHT, Technical University of Denmark 5205, Lyngby, Denmark; The Pennsylvania State University, University Park, Pennsylvania, USA

**Keywords:** *Pseudomonas putida*, transcriptional regulation, metabolism, PQQ, synthetic metabolism, one-carbon assimilation, metabolic engineering, synthetic biology, alcohol, aldehyde

## Abstract

*Pseudomonas* species display exceptional metabolic versatility that underpins their ecological success and broad relevance for synthetic biology, environmental microbiology, bioremediation, and bioproduction. A central contributor to this versatility is the *ped* gene cluster, which encodes pyrroloquinoline quinone (PQQ)-dependent dehydrogenases that catalyze the oxidation of a wide range of alcohols and aldehydes. These enzymes support both assimilation and detoxification processes with high catalytic efficiency. This review compiles current knowledge on genetic organization, enzymatic functions, and multi-level regulation of the *ped* cluster, with a focus on *Pseudomonas putida* KT2440 and *Pseudomonas aeruginosa* PAO1. The roles of regulatory components [e.g., the iron (Fe^2+^)-dependent YiaY dehydrogenase and the hybrid PP_2683 histidine kinase] are examined for their capacity to respond to short-chain alcohols through a complex signal transduction network. Additional genetic elements, including *pedF* and *pedG*, along with poorly characterized open reading frames (e.g., *pedD*, *PP_2666*, and *PP_2678*), which support enzymatic maturation, electron flow, and modulation of surface-associated behaviors are likewise considered. Comparative analysis across the *Pseudomonas* genus showed that *ped*-like clusters are conserved but display substantial differences in gene content and arrangement, suggesting adaptations to specific ecological contexts. We evaluate these elements in detail to define a reference framework for future mechanistic studies. By bringing together functional and regulatory features of the cluster, our article provides a basis for exploiting the Ped system as a modular platform in applied microbiology. This integrated view aims to guide ongoing and future fundamental and applied research on alcohol oxidation in gram-negative bacteria.

## INTRODUCTION

The genus *Pseudomonas* comprises a phylogenetically diverse group of gram-negative, rod-shaped bacteria characterized by metabolic versatility and wide ecological presence (1). These bacteria are found in terrestrial and aquatic environments, plant-associated habitats, and clinical contexts (2). *Pseudomonas* species contribute to multiple biological processes and have been used in industrial applications due to their remarkable physiological adaptability (3). *P. aeruginosa*, for instance, is known for its role as an opportunistic human pathogen (4). In contrast, *P. fluorescens* and *P. putida* have been extensively used in environmental cleanup, plant growth enhancement, and industrial biocatalysis (5–8). The capacity of *Pseudomonas* species to degrade complex organic compounds, produce various secondary metabolites, and survive in adverse conditions across oxygen (O_2_) gradients underscores their relevance in medical, environmental, and agricultural applications (9, 10).

**Fig 1:**
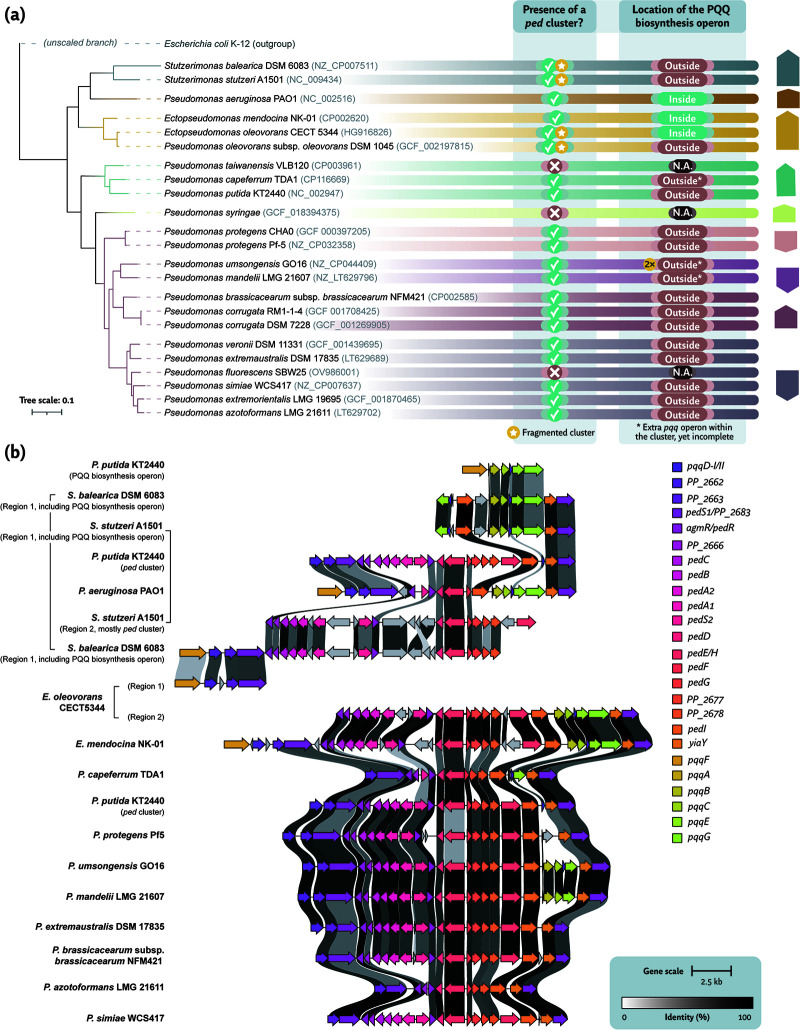
Taxonomic analysis and genomic conservation of the *ped* cluster across *Pseudomonas* species. (**a**) Distribution of the *ped* cluster and associated *pqq* biosynthesis genes in species from the *Pseudomonas*, *Stutzerimonas*, and *Ectopseudomonas* genera. Incomplete *pqq* operons located within the *ped* cluster are indicated with an asterisk. Taxonomic groups and subgroups are color-coded. Lineages lacking the cluster, including *P. resinovorans*, *P. chlororaphis*, *P. koreensis*, and *P. jessenii*, are not shown. The phylogenetic tree was constructed using autoMLST (23). (**b**) Synteny and conservation of the *ped* cluster and co-regulated *pqq* operon in selected *Pseudomonas* species, aligned to *P. putida* KT2440 as reference. For gene-level annotations, please refer to Table 1. Visualization generated using clinker and clustermap.js (24). N.A., not applicable.

The *ped* cluster encodes a key metabolic module that enables *Pseudomonas* to exploit alcohols and aldehydes as carbon sources, detoxification targets, and environmental signals, thereby supporting ecological fitness and underlying the biotechnological relevance of these species (Fig. 1). This conserved genomic region encodes multiple alcohol and aldehyde dehydrogenases that connect peripheral substrate oxidation to central and secondary metabolism. The name of the cluster reflects its original association with the 2-phenylethanol degradation pathway (11). Pyrroloquinoline quinone (PQQ)-dependent alcohol dehydrogenases (ADHs), including PedE and PedH in *P. putida* KT2440 (12), are among the enzymes encoded in the *ped* cluster. These dehydrogenases are endowed with broad substrate specificity and catalyze the oxidation of various alcohols and aldehydes with higher catalytic activities due to their favorable thermodynamics, compared to NADH-dependent counterparts. Moreover, such broad-range oxidative activities support metabolic flexibility in diverse environmental contexts (12). Besides ADHs, the *ped* cluster also encodes regulatory and biochemical elements required for the expression, maturation, and activation of the PQQ-dependent ADHs. Notably, PQQ biosynthesis requires O_2_ (13–15), although some bacteria can acquire PQQ from external sources under anoxic conditions (16). The products of this cluster also control PQQ biosynthesis and environmental signal integration (17–19), including the sensing of rare earth elements (REEs; [20–22]).

The *ped* cluster is regulated at three distinct levels, and these features contribute to its functional complexity (Table 1). The Ped system in *Pseudomonas* species shares several traits with REE-responsive alcohol oxidation systems found in methanotrophic and methylotrophic bacteria. One of the best-characterized Ped systems is found in *Methylobacterium extorquens* AM1 (25, 26). In some *Pseudomonas* strains, lanthanide (Ln^3+^)-dependent dehydrogenases can catalyze methanol oxidation (27–30), and the metabolic potential encoded by the *ped* cluster has been exploited for bioremediation, synthetic methylotrophy, and ethylene glycol assimilation (31–33).

**TABLE 1:** Members of the *ped* cluster of *P. putida* KT2440, including their predicted function, cellular localization, and closest homolog in the well-characterized *P. aeruginosa* PAO1

PP#	Gene name	Function^*a*^	Cell localization^*b*^	PAO1 homolog^*c*^	UniProt ID
PP_2662	–^*d*^	(Porin domain-containing protein)	(Outer membrane)	PA1974 (QC 99%; ID 72%)	Q88JI7
PP_2663	–	(GfdT, nitric oxide-sensing protein)	(Cytoplasmic)	PA1975 (*nosP*) (QC 100%; ID 74%)	Q88JI6
PP_2664	*pedS1*	Hybrid histidine kinase	(Cytoplasmic membrane)	PA1976 (*ercS*’, *nahK*) (QC 98%; ID 75%)	Q88JI5
PP_2665	*agmR* (*pedR1*)	LuxR-type transcriptional activator	(Cytoplasmic)	PA1978 (*erbR*) (QC 100%; ID 86%)	Q88JI4
PP_2666	*–*	(Rhodanese domain-containing protein)	(Non-cytoplasmic)	N.D.	Q88JI3
PP_2667	*pedC*	Transport permease protein	Cytoplasmic membrane	N.D.	Q88JI2
PP_2668	*pedB*	ABC efflux transporter, ATP-binding protein	Cytoplasmic membrane	N.A.	Q88JI1
PP_2669	*pedA2*	PQQ-dependent catabolism-associated β-propeller protein	(Non-cytoplasmic)	N.D.	Q88JI0
PP_5538	*pedA1*	ABC transporter, substrate-binding protein	(Non-cytoplasmic)	N.D.	A0A140FW92
PP_2671	*pedS2*	Histidine kinase	Cytoplasmic membrane	PA1979 (*eraS/exaD*) (QC 48%; ID 54%)	Q88JH8
PP_2672	*pedR2* (*exaE*)	Transcriptional activator protein	Cytoplasmic	PA1980 (*eraR*) (QC 100%; ID 66%)	Q88JH7
PP_2673	*pedD*	(Pentapeptide repeat family protein)	(Extracellular)	PA1981 (QC 94%; ID 63%)	Q88JH6
PP_2674	*pedE* (*qedH-I*)	Quinoprotein alcohol dehydrogenase	Periplasmic	PA1982 (*exaA*) (QC 100%; ID 84%)	Q88JH5
PP_2675	*pedF*	Cytochrome *c*_550_	(Periplasmic)	PA1983 (*exaB*) (QC 99%; ID 64%)	Q88JH4
PP_2676	*pedG*	(Periplasmic solute-binding protein)	(Periplasmic)	N.D.	Q88JH3
PP_2677	–	(Quinoprotein dehydrogenase-associated SoxYZ-like carrier)	(Non-cytoplasmic)	N.D.	Q88JH2
PP_2678	–	(SoxH/quinoprotein relay system zinc metallohydrolase)	(Non-cytoplasmic)	N.D.	Q88JH1
PP_2679	*pedH* (*qedH-II*)	Quinoprotein ADH	Periplasmic	PA1982 (*exaA*) (QC 99%; ID 52%)	Q88JH0
PP_2680	*pedI* (*aldB-II*)	NAD^+^-dependent aldehyde dehydrogenase	Cytoplasmic	PA1984 (*exaC*) (QC 100%; ID 88%)	Q88JG9
PP_2681	*pqqD-II*	PqqA binding protein	Cytoplasmic	PA1988 (*pqqD*) (QC 100%; ID 51%)	Q88JG8
PP_2682	*yiaY*	Fe-containing ADH	Cytoplasmic	PA1991 (*ercA*) (QC 100%; ID 87%)	Q88JG7
PP_2683	(*yiaZ*)	Hybrid histidine kinase	(Cytoplasmic membrane)	PA1992 (*ercS*) (QC 98%; ID 72%)	Q88JG6

^*a*^ Predicted function based on UniProt and InterPro annotations, indicated in parentheses.

^*b*^ Predicted localization based on PSORTb (v.3.0.2) (34) and SignalP (35) analyses; proteins predicted in multiple compartments (excluding the cytoplasm) are classified as “non-cytoplasmic”; predictions are indicated in parentheses.

^*c*^ N.D., not determined; N.A., not applicable (assigned when multiple hits were returned with low sequence identity); QC, query cover; ID, identity.

^*d*^ –, not applicable.

Most studies on the Ped system published so far have focused mainly on *P. putida* KT2440 and, to a lesser extent, *P. aeruginosa* PAO1 and PA14. However, several PQQ-dependent ADHs have also been identified in other isolates (Table 1). These include *P. protegens* Pf-5, *P. putida* strains HK5, DOT-T1E, and U, *Pseudomonas* spp. W1 and BB1, *P. extremaustralis*, and *P. testosteroni* (36–44). Indeed, a comparative analysis using the *Pseudomonas* Genome Database (https://www.pseudomonas.com/ [45]) with the conserved regulators of the cluster YiaY and PP_2683 as reference queries identified more than 1,000 strains from over 70 *Pseudomonas* species that potentially encode a homolog of the *ped* cluster. Several of these species are relevant to biotechnology. Notable examples include strains of *P. protegens* (e.g., Pf-5), *P. fluorescens*, *P. corrugata*, *Ectopseudomonas oleovorans* (previously known as *P. pseudoalcaligenes*, e.g., strain CECT5344), *P. putida* (e.g., strain DOT-T1E), *Stutzerimonas stutzeri* (previously *P. stutzeri*), *P. oleovorans*, *P. resinovorans*, and *P. denitrificans*, along with the extensively studied *P. putida* KT2440 and *P. aeruginosa* PAO1 (Fig. 1). *P. protegens* and *P. fluorescens* are primarily applied as biocontrol agents and for plant growth promotion (46). *P. corrugata* also supports plant protection (47), although some strains are recognized as potentially phytopathogenic. *E. oleovorans* CECT5344 has been used in bioremediation (48), especially for detoxifying cyanide-rich industrial wastewater (49). As indicated above, *P. putida* serves as a model for biotransformation and degradation of aromatic hydrocarbons and solvents (50). In particular, *P. putida* KT2440 is widely applied as a host for synthetic biology and metabolic engineering (51–53). *S. stutzeri* is recognized for denitrification and nitrogen cycling in wastewater treatment (54). *P. oleovorans* and *P. resinovorans* are involved in hydrocarbon degradation and polyhydroxyalkanoate (PHA) production (55). *P. denitrificans* is used in the industrial production of vitamin B_12_ (cobalamin [56]), and *P. aeruginosa*, although identified as an opportunistic pathogen (57), has also been employed for biosurfactant synthesis and bioremediation (58).

Interestingly, the *ped* cluster displays a largely conserved genomic organization across these species (Fig. 1b). Individual gene components are missing in some cases, for example, in *P. aeruginosa* and *P. capeferrum* TDA1 (59). In other species, including *E. oleovorans* CECT5344 and *S. stutzeri* A1501, the cluster is fragmented, with genes distributed across distant chromosomal regions. The *pqq* biosynthesis operon is present in all *Pseudomonas* species carrying the *ped* cluster. In several genomes, additional *pqq* operons are detected. These secondary operons are often incomplete and can be located either within the *ped* cluster or elsewhere in the genome (Fig. 1b), suggesting that PQQ biosynthesis relies on a combination of core and auxiliary genetic elements.

This article presents and discusses relevant findings on the functional roles of individual components of the *ped* cluster and the interactions among these gene products. This functional overview provides the basis for examining how these components are coordinately regulated and integrated into broader metabolic networks. The text then addresses differences in regulatory strategies and evolutionary trajectories across *Pseudomonas* species. *P. putida* KT2440, the most studied species representative, serves as a reference framework throughout. Emphasis is placed on metabolic activity, structural organization, and regulatory pathways. The analysis also identifies key gaps in understanding that currently limit the rational engineering of this system for synthetic biology, environmental microbiology, bioremediation, and bioproduction.

## A SPOTLIGHT ON EACH MEMBER OF THE GENE CLUSTER

**Fig 2:**
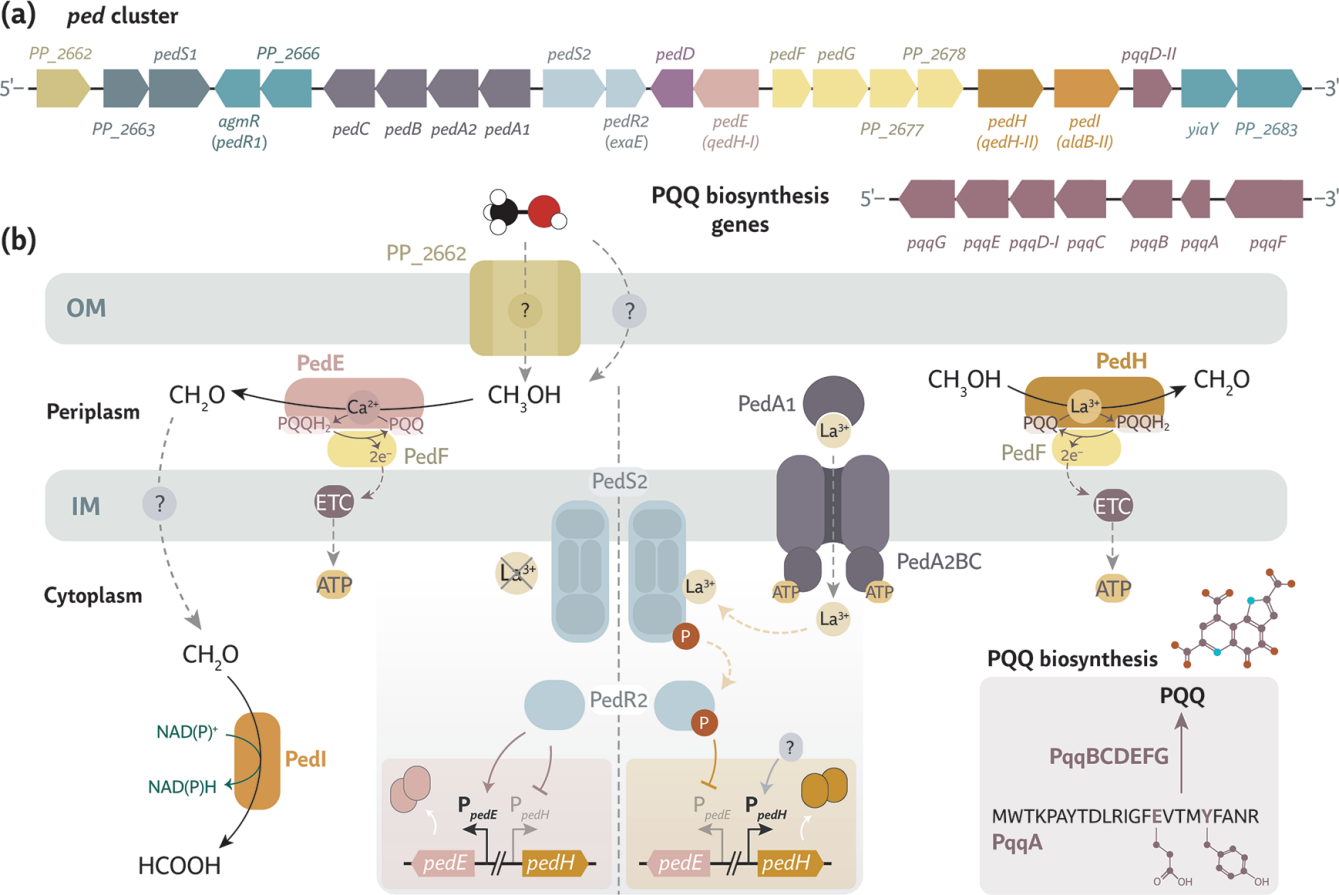
Genetic organization (**a**) and functional roles (**b**) of characterized members of the *ped* cluster of *P. putida* KT2440 involved in alcohol processing, using methanol as a representative substrate. The figure includes genes responsible for PQQ biosynthesis, alcohol and aldehyde oxidation, and REE (e.g., La^3+^) transport and sensing through the two-component system PedS2-PedR2. Genes are not drawn to scale. Abbreviations: OM, outer membrane; IM, inner membrane; ETC, electron transfer chain.

In this section, each gene of the *ped* cluster in *P. putida* KT2440 is examined within the context of its genomic organization and functional grouping, including transporters, regulators, alcohol and aldehyde dehydrogenases, and enzymes required for maturation and activation of these ADHs. Functional insights are integrated and compared across *Pseudomonas* strains to highlight conserved architectures, variable gene content, and strain-specific adaptations. This perspective addresses how differences in gene arrangement, presence or absence of specific components, and cluster fragmentation may reflect ecological specialization, regulatory integration, or horizontal gene transfer events. The genetic organization of the cluster is illustrated in Fig. 2 and Table 1 summarizes biochemical functions, subcellular localization, and homology with genes in *P. aeruginosa* PAO1.

### Transporters

#### The *PP_2662* porin

*PP_2662* encodes a putative outer membrane porin believed to mediate the transport of short-chain alcohols. Functional data on this gene are limited, but experimental findings support its role in alcohol uptake and stress adaptation. For instance, during adaptive laboratory evolution of *P. putida* KT2440 on ethylene glycol, truncation of *PP_2662* was observed. This deleterious modification likely reduced the accumulation of toxic oxidation intermediates in slow-growing cells and removed a metabolic bottleneck. Glycolate accumulation ceased after truncation, and growth improved substantially. These effects were confirmed through reverse engineering of the key mutations (31) found upon adaptive laboratory evolution (60).

*PP_2662* expression also increased sharply upon rehydration of desiccated *P. putida* KT2440 cells (61). This observation suggests involvement in the transport and catabolism of polyhydric alcohols accumulated during desiccation. Structural studies indicated that porins typically form trimers with channels wide enough to accommodate glucose-sized molecules (62), supporting the hypothesis that short-chain alcohols can permeate through *PP_2662*. Furthermore, expression of *PP_2662* is induced along with other *ped* genes in response to ethanol and methanol (28). This co-expression pattern aligns with its proposed role in alcohol transport and metabolism.

#### The PedA1A2BC lanthanide transporter

*P. putida* KT2440 can use REEs from the lanthanide series (e.g., La^3+^) as cofactors for alcohol oxidation (12, 63). PedH, a PQQ-dependent periplasmic ADH, requires Ln^3+^, whereas its calcium (Ca^2+^)-dependent counterpart, PedE, does not (12). PedH has higher activity than PedE and shows enhanced performance compared to NAD(P)^+^-dependent ADHs, likely due to favorable reaction thermodynamics (12, 64). Expression of *pedE* or *pedH* depends on Ln^3+^ availability, a phenomenon known as the *REE switch (*12, 65). For the PedS2-PedR2 system to detect Ln^3+^ ions and initiate the switch, these charged species must reach the cytoplasm (Fig. 2b).

In the methylotroph *M. extorquens* AM1, the TonB-dependent receptor LutH transports Ln^3+^ into the periplasm (66), where Ln^3+^-dependent methanol dehydrogenases (MeDHs) are typically located (67). *P. putida* KT2440 lacks a LutH homolog, and its outer membrane transport mechanism remains unresolved. Potential alternatives include non-specific metal transporters for Fe^2+^/Fe^3+^ ions, low-affinity porins, or yet unidentified systems.

Once in the periplasm, translocation of Ln^3+^ into the cytoplasm is mediated by the PedA1A2BC system. This ATP-binding cassette (ABC) transporter complex has been shown to be essential for PedH-dependent growth (e.g., in a ∆*pedE* genetic background [27]). *P. aeruginosa* PAO1 lacks this transporter as it does not have a REE-dependent dehydrogenase (Fig. 1), while *M. extorquens* AM1 encodes a similar system (27, 67). The REE switch depends on PedA1A2BC and the surrounding ionic environment. Competing metal ions such as Fe^2+^/Fe^3+^, copper (Cu^2+^), or zinc (Zn^2+^) can reduce Ln^3+^ availability and impair transporter function. In a ∆*pedE* strain, for instance, excess Fe^2+^ (as FeSO_4_) inhibited growth unless lanthanide levels were increased (27). This effect was not reversed by deleting pyoverdine, suggesting mismetallation rather than uptake competition. Fe^2+^/Fe^3+^ may displace La^3+^ from binding sites, disrupting PedS2 signaling or PedH activity. Conversely, La^3+^ can inhibit Fe^2+^/Fe^3+^ uptake through non-siderophore pathways, further limiting cellular function (27).

Importantly, not all REEs elicit equivalent physiological effects. Light lanthanides, for example, lanthanum, cerium (Ce^3+^), and neodymium (Nd^3+^), support growth of *P. putida* KT2440 at nanomolar levels (68), while praseodymium (Pr^3+^) increases PedH activity *in vitro* (12). Addition of heavier lanthanides to the culture medium does not enhance bacterial growth and may be inhibitory, likely due to mismetallation as well (68). Transcriptomic studies show that regulators PedS2-PedR2 and PedH respond differently to various lanthanides, with weak or entirely absent activation by heavier ones (68). Although some organisms, for example, *Methylobacterium*, can use samarium (Sm^3+^), europium (Eu^3+^), or gadolinium (Gd^3+^), these elements are less effective as effectors when compared to light lanthanides (21).

### Alcohol and aldehyde dehydrogenases

#### The PQQ-dependent alcohol dehydrogenases PedE and PedH

The PQQ-dependent alcohol dehydrogenases PedE and PedH are the main catalytic enzymes encoded by the *ped* gene cluster and mediate alcohol oxidation in *P. putida* KT2440. These enzymes show broad substrate specificity and oxidize various primary and secondary alcohols, including methanol, ethanol, 1-butanol, 1-hexanol, and 2-phenylethanol, as well as short-chain aldehydes. These activities have been both demonstrated *in vivo* and *in vitro* (12, 17). Whereas both PedE and PedH use PQQ as a redox cofactor, PedE depends on Ca^2+^ ions, and PedH requires trivalent lanthanides (Ln^3+^) as essential cofactors. These metal ions coordinate with the PQQ molecule at the catalytic site and regulate enzymatic activity (Fig. 2). Several *Pseudomonas* species, including *P. aeruginosa* and *P. capeferrum* TDA1 (18), encode only the Ca^2+^-dependent PedE homolog (Fig. 1).

PedE and PedH share 52% sequence identity and have homologs in methylotrophic and methanotrophic bacteria, including XoxF and MxaF variants in *M. extorquens* AM1 and *M. capsulatus* Bath, with identities above 35%. Indeed, overexpression of *pedE* and *pedH* enables synthetic methylotrophy by supporting methanol oxidation prior to assimilation in *P. putida* KT2440 (32). Nonetheless, the closest homolog of PedE and PedH in *M. extorquens* AM1 is ExaF (*MexAM1_META1p1139*), a Ln^3+^-dependent methanol and ethanol dehydrogenase, which elicits formaldehyde oxidation (69). Since PedE and PedH efficiently oxidize short-chain aldehydes such as acetaldehyde and butyraldehyde, they may also oxidize formaldehyde (69, 70).

These enzymes feature a rare disulfide ring near the PQQ-binding site with functional significance. In ExaA from *P. aeruginosa*, adjacent cysteine residues Cys105 and Cys106 have been linked to electron transfer from reduced PQQH_2_ to the electron acceptor cytochrome *c*_550_ (71). This disulfide motif is conserved across homologs from *Methylobacterium*, *Comamonas testosteroni*, *Paracoccus denitrificans*, and other pseudomonads, for example, *P. putida* HK5 (71–77). The three-dimensional crystal structure of PedH has been resolved and provides further insight into its catalytic architecture (78).

In conclusion, PedE and PedH exemplify the enhanced catalytic, thermodynamic, and physiological capabilities of PQQ-dependent ADHs, combining broad substrate scope, high oxidative efficiency, and direct coupling to respiratory electron transfer that distinguish them from classical NAD(P)H-dependent alcohol dehydrogenases.

#### The aldehyde dehydrogenase PedI

PedI, also known as AldB-II, is a conserved aldehyde dehydrogenase that uses NAD(P)^+^ as an electron acceptor and catalyzes the oxidation of diverse aldehydes (32, 37). The *pedI* gene is among the most strongly upregulated in *P. putida* KT2440 after exposure to alcohols such as methanol and ethylene glycol. This pattern indicates a probable role in oxidizing aldehydes generated during alcohol metabolism (11, 28).

Despite the strong transcriptional response, deletion of *pedI* does not result in severe phenotypic changes under alcohol stress. Inactivation studies showed limited impact on methanol tolerance (28). RB-*Tn*Seq analyses also revealed no significant growth defects on short-chain alcohols when the gene was disrupted (17). These results suggest functional redundancy. *P. putida* and other pseudomonads are naturally tolerant to reactive aldehydes and possess multiple aldehyde dehydrogenases encoded in their genomes (79, 80). Further biochemical studies are needed to define specific metabolic functions of PedI, including its cofactor specificity and substrate turnover kinetics. Yet, its repeated overexpression in alcohol exposure conditions supports a role in detoxification of aldehyde intermediates and protection from associated cellular damage.

### Maturation and activation

#### The pentapeptide-repeat protein PedD

PedD is an uncharacterized member of the pentapeptide repeat protein family and is conserved across both methylotrophic and non-methylotrophic bacteria, including *Pseudomonas* species. Although its physiological role remains undefined, a truncation mutation in *P. putida* U impaired growth in the presence of several short-chain alcohols, suggesting a functional connection to alcohol metabolism (37). The expression of *pedD* follows the transcriptional upregulation pattern of the *ped* cluster in response to multiple alcohol substrates (28), indicating co-regulation within this metabolic network.

Pentapeptide repeat proteins contain repetitive sequence motifs that fold into right-handed quadrilateral β-helix structures, which resemble DNA helices. These structures facilitate interactions with nucleic acid-binding proteins, including DNA gyrases, as shown for McbG in *Escherichia coli* (81, 82). Some proteins in this group, for example, MfpA in *Mycobacterium tuberculosis* and Qnr in *Klebsiella pneumoniae*, contribute to fluoroquinolone resistance by mimicking DNA and blocking antibiotic binding to DNA gyrase (83). Additional functions have been proposed, including structural roles in biofilm matrices (84). Speculative roles such as metal ion scavenging or chelation have not been tested. Based on its predicted extracellular localization and transcriptional induction by alcohols, PedD may serve noncanonical functions within the *ped* cluster that relate to biofilm physiology, consistent with other cluster members. Biochemical and genetic studies are needed to define its molecular targets and physiological relevance.

#### PedF, a cytochrome *c*_550_ component

PedF is a periplasmic cytochrome *c*_550_-type protein essential for maintaining the activity of the PQQ-dependent alcohol dehydrogenases PedE and PedH. PedF reoxidizes the reduced PQQ cofactor (Fig. 2), and its functional relevance is supported by the severe growth defects observed in *P. putida* KT2440 and *P. putida* U upon gene truncation during growth on short-chain alcohols (17, 37). PedF shares high sequence identity with cytochrome *c*_L_ of *M. extorquens* AM1 and ExaB of *P. aeruginosa* PAO1. Both proteins are well-characterized periplasmic electron carriers involved in alcohol metabolism (85, 86).

The terminal electron acceptor for PedF in *P. putida* remains unknown. It is proposed that PedF transfers electrons to terminal cytochromes *c* in the respiratory electron transport chain. In methylotrophs, for example, *M. extorquens* AM1, electrons pass from cytochrome *c*_L_ to cytochrome *c*_H_ and then to a terminal cytochrome oxidase (85–89). *P. putida* and *P. aeruginosa* do not possess homologs of cytochrome *c*_H_, which indicates a divergence in electron transfer pathways between methanol-utilizing methylotrophs and non-methylotrophic pseudomonads (90).

Transcriptomic data indicates a connection between PedF-mediated electron transfer and cytochrome *c* oxidase activity. The genes encoding the heme-copper *cbb_3_*-type cytochrome *c* oxidase complex (*ccoNOQP*, *PP_4255-PP_4258*) were strongly upregulated in *P. putida* KT2440 exposed to methanol stress (28). These *cbb_3_*-type oxidases are prevalent in Proteobacteria and display structural and functional characteristics that differ from canonical cytochrome *c* oxidases (91). In *Stutzerimonas stutzeri* (previously *Pseudomonas stutzeri*), for example, the *cbb_3_* complex transfers two protons per electron pair and contributes to O_2_ scavenging, redox control, and ion homeostasis in microaerobic environments (91, 92). Their high O_2_ affinity enables respiration under micro-oxic or hypoxic conditions, and their expression is commonly linked to general stress responses across bacterial taxa (28). Whether the induction of *cbb_3_* oxidases during alcohol stress serves a protective function or reflects a metabolic adaptation within the PedF-dependent electron transfer system remains unresolved.

#### Periplasmic binding-protein PedG

PedG is predicted to function as a periplasmic binding protein structurally related to the substrate-binding components of ABC importers, although the associated transporter is yet to be identified. Despite low sequence identity, structural homologs of PedG have been described in methylotrophic and methanotrophic bacteria. These include XoxJ and MxaJ in *M. extorquens* PA1 and AM1, MoxJ in *P. denitrificans*, and MxaJ in *Acetobacter methanolicus*. All these candidates display a root mean square deviation < 4 Å and are essential for the function of their corresponding MeDHs (67, 93–95).

Multiple functional roles have been proposed for these proteins. These include regulatory activity, assistance in enzyme maturation, or modulation of interactions between dehydrogenases and cytochromes (93–96). Recent studies suggest that PedG orthologs in methylotrophic bacteria such as XoxJ and MxaJ may bind partially folded apo-MeDH. This binding facilitates the insertion of PQQ and La^3+^ or Ca^2+^, which is sterically hindered in the mature enzyme conformation (96, 97). Deletion of *xoxJ* compromises XoxF function and impairs methylotrophic growth under Ln^3+^-free conditions. Notably, expression of the Ca^2+^-dependent *mxa* operon does not restore methanol assimilation in these mutants, indicating that XoxJ may exert post-transcriptional control on the MxaFI system (67, 96).

*P. aeruginosa* PAO1 lacks a *pedG* homolog within the *ped* cluster. However, structurally similar proteins located in other regions, such as PA_1604, which is putatively linked to nicotinate dehydrogenase, may perform comparable functions, possibly in coordination with ExaAB. In *M. extorquens* AM1, the *exaF* locus is flanked by genes encoding PedG- and PedF-like proteins (*META1p1138* and *META1p1137*), which supports evolutionary and functional conservation of this module (70).

PedG has been annotated *in silico* as a member of the PAAT family of amino acid ABC transporter substrate-binding proteins. Nonetheless, functional genomics data from transposon libraries show only modest growth defects after *pedG* disruption. These effects are less severe than those observed for *pedF*, suggesting redundancy or functional compensation by paralogs (17). This finding supports the possibility that, as in *P. aeruginosa* PAO1, proteins outside the *ped* cluster may support PedE- or PedH-dependent functions.

#### Quinoprotein dehydrogenase-associated proteins PP_2666, PP_2677, and PP_2678

Three open reading frames within the *ped* cluster of *P. putida* KT2440—that is, PP_2666, PP_2677, and PP_2678—are absent in *P. aeruginosa* but conserved in methylotrophic bacteria and other Proteobacteria encoding Ln^3+^- and PQQ-dependent ADHs (98). *PP_2666* encodes a predicted rhodanese-domain protein; *PP_2677* encodes a fused SoxYZ-like protein involved in sulfur metabolism (99). *PP_2678* encodes a SoxH-like Zn^2+^-dependent metallo-hydrolase. These three proteins are consistently associated with systems that encode PQQ-dependent ADHs containing adjacent cysteine residues in the active site. This structural motif has been implicated in electron transfer to cytochrome *c*_550_ (71–75).

Although these disulfide bridges are also present in Ca^2+^-dependent enzymes, for example, PedE and ExaA, their function in bacterial species that lack PP_2666 to PP_2678, including *P. aeruginosa*, remains unclear (Table 1). In *M. extorquens* AM1, homologs of these genes (i.e., *META1p1703*, *META1p1704*, and *META1p1705*) are co-transcribed within an operon located near the REE-dependent *xoxFGJ* locus, along with a thioredoxin-like gene. This gene context strongly suggests a conserved role in enzyme maturation or redox control.

Random-barcode transposon sequencing (RB-*Tn*Seq) showed minimal phenotypic effects when these genes were disrupted in *P. putida* KT2440 grown on various short-chain alcohols. These experiments were performed in REE-deficient conditions, where the Ca^2+^-dependent PedE is likely the dominant ADH (17). These findings suggest that the role of PP_2666 to PP_2678 becomes relevant under Ln^3+^-utilizing conditions. These proteins may contribute to disulfide bridge formation or cofactor stabilization in REE-dependent ADHs. An alternative hypothesis proposes that these proteins protect PQQ from nucleophilic attack by sulfur-containing species, for example, sulphones (100). In *Hyphomicrobium denitrificans*, thiosulfate supplementation combined with auxiliary electron donors inhibited methanol-based growth but not formate-based growth. This outcome was likely due to the formation of PQQ-sulfur adducts that interfere with ADH activity (101).

### Regulators

#### PP_2663 and PedS1 as regulators of swarming and biofilm dispersal

**Fig 3:**
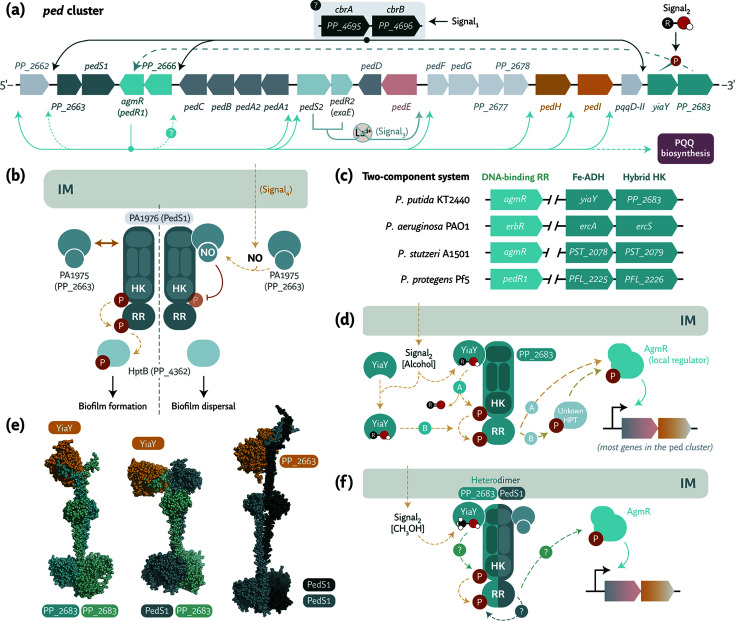
Proposed regulation patterns of the *ped* cluster in *P. putida* KT2440. (**a**) Multi-layered regulation of the cluster based on several signals, potentially originating from the two-component system (TCS) CbrAB, which may activate the expression of *yiaY*, *PP_2683*, and *agmR*. Upon activation, these individual regulators are proposed to induce transcription of the other cluster genes. The final regulatory layer involves the TCS PedS2-PedR2, which responds to REEs by repressing *pedE* expression. (**b**) Regulation of the TCS PA_1975–PA_1976 in *P. aeruginosa* PAO1, homologous to PedS1-PP_2663 in *P. putida* KT2440, where NO sensing leads to modulation of the histidine kinase (HK) PA_1976, anchored to the inner membrane (IM), through direct interaction. In this configuration, NO sensing ultimately results in biofilm swarming via other response regulators (RR). Orthologs in *P. putida* KT2440 are noted in parentheses. (**c**) Conservation of the TCS genes *PP_2683* and *agmR*, along with the Fe^2+^-containing ADH gene *yiaY*, among pseudomonads. (**d**) Putative model for activation of the TCS formed by PP_2683 and AgmR, potentially modulated by YiaY activity through either binding to or oxidation of short-chain alcohols. The potential involvement of a histidine phosphotransferase protein (Hpt) remains to be confirmed. This model is informed by experimental data and partial conservation with the known regulatory patterns indicated in panel (**b**). (**e**) AlphaFold 3 (110)-predicted protein structures of PP_2683 with YiaY, a heterodimer of PP_2683-PedS1 with YiaY, and PedS1 homodimers with PP_2663. (**f**) Hypothesized heterodimer formation between PedS1 and PP_2683 may lead to differential activation of the cluster depending on the short-chain alcohol supplemented to the medium (111). A conceptual model illustrating this heterodimer-dependent regulatory pattern is shown in the diagram.

The co-cistronic genes *PP_2663* and *PP_2664* encode a putative nitric oxide (NO)-binding protein and a soluble hybrid histidine kinase (HHK), PedS1, respectively. These genes share homology with the *nosP-nahK* (*PA_1975–PA_1976*) locus of *P. aeruginosa* PAO1 (Fig. 3b, Table 1). In this bacterium, the NosP-based signaling cascade promotes biofilm dispersal in response to sub-micromolar NO concentrations (102). Typically, HHKs relay phosphate groups to histidine-containing phosphotransfer proteins (HPTs; [103]). NosP modulates the activity of NahK, which phosphorylates HptB (PA_3345). The signal is then passed to the global regulator RsmA (104, 105). HptB homologs exist in *P. putida* KT2440, including *PP_4362*, although this gene is deleted in genome-reduced strains, for example, *P. putida* EM42 (106, 107). *P. putida* KT2440 also encodes RsmA (PP_4472), RsmE (PP_3832), and RsmI (PP_1746), which inhibit translation of mRNAs related to biofilm formation and quorum sensing. Deleting these genes reduced swimming and swarming and enhanced biofilm formation (108), suggesting that Rsm proteins promote motility and a planktonic lifestyle. NO-mediated regulation of biofilms is conserved in *Burkholderia thailandensis*, *Shewanella oneidensis*, *Vibrio cholerae*, and *Legionella pneumophila* (102, 109). These findings suggest that PP_2663 and PedS1 could regulate swarming via NO sensing, rather than (or in addition to) sensing alcohols.

Ethanol has also been implicated in swarming in *P. aeruginosa* PA14 via the *ped* cluster. However, whether this response involves NosP–NahK or another regulatory system is unclear (112). ErdR (PA14_17670) is a key regulator of ethanol-induced swarming (112). PedS1 may form heterodimers with PP_2683, another HHK, possibly producing different signaling outputs. This possibility is discussed in the following section.

#### Iron-containing ADH YiaY and its co-cistronic hybrid histidine kinase PP_2683

Co-cistronic genes *yiaY* and *PP_2683*, the latter recently designated *yiaZ*, have emerged as conserved regulatory elements that influence short-chain alcohol metabolism in *Pseudomonas* species. The *yiaY* gene encodes a Fe^2+^-dependent alcohol dehydrogenase, whereas *PP_2683* encodes a histidine kinase with features characteristic of sensory transduction systems. *Zymomonas mobilis* ZM4 contains an ortholog of YiaY, whose crystal structure has been resolved. This enzyme catalyzes the oxidative reaction under aerobic conditions (113). Disruption of either gene in *P. putida* or *P. aeruginosa* causes extended lag phases and impaired growth when grown on short-chain alcohols (17–19, 28, 114).

Inactivation of either *yiaY* or *PP_2683* results in transcriptional repression of the *ped* cluster. This repression mechanism includes *pedF*, *agmR*, the PQQ-dependent alcohol dehydrogenase genes, and the *pqq* biosynthetic operon. These findings suggest that this module acts upstream of the main regulatory network (Fig. 3a) (18, 19, 32, 111). Although the mechanism remains unresolved, AlphaFold3-based structural modeling (110) predicts a direct interaction between the *N*-terminal domains of YiaY and PP_2683 (Fig. 3e). This *in silico* result points to a possible coordinated sensory and response complex that depends on YiaY function (Fig. 3d).

Recently, overexpression of *yiaY* and *PP_2683* has been harnessed to engineer synthetic methylotrophy in *P. putida*. Synthetic serine cycles were coupled with efficient methanol oxidation mediated by either PedE or PedH depending on REE availability (32). This overexpression strategy appears to prime the cells for faster induction of alcohol catabolic pathways (32, 111).

Whether alcohol sensing occurs directly through PP_2683 or involves redox coupling with YiaY remains unknown. Mutation of the conserved NAD(P)^+^-interacting residue H281 in YiaY impairs cluster induction, indicating a catalytic or structural role in signal transmission (111). Current evidence supports a model in which YiaY and PP_2683 regulate AgmR activation, which in turn promotes expression of the *ped* cluster and PQQ biosynthetic genes (Fig. 3a and d).

#### The local AgmR regulator

AgmR, also known as PedR1 or ErbR, was first identified in *P. aeruginosa* as a transcriptional regulator of glycerol metabolism (115). A homolog was found in *P. putida* ATCC 11172 using mini-Tn*5* mutagenesis. Disruption of the gene impaired growth on glycerol and ethanol and caused a sharp decline in PQQ-dependent ADH activity (116). This observation identified AgmR as a central regulator of the *ped* cluster. Regulatory studies across *Pseudomonas* species revealed a complex hierarchy of signaling pathways and two-component systems (TCS; [18, 19, 37, 114, 117, 118]). AgmR activates the transcription of both the core genes of the *ped* cluster and the separately encoded PQQ biosynthetic operon *pqqFABCDE* in *P. putida* KT2440 (details of the PQQ biosynthetic genes are provided in Table 2 and Fig. 1 and 2).

**TABLE 2:** PQQ biosynthesis genes and other regulatory genes distal to the *ped* gene cluster

PP#	Gene name	Function^*a*^	Cell localization^*b*^	PAO1 homolog^*c*^	UniProt ID
Biosynthesis genes					
PP_0375	(*pqqG*)	Prolyl oligopeptidase family protein	Cytoplasmic	PA1990 (*pqqG*) (QC 97%; ID 52%)	Q88QV9
PP_0376	*pqqE*	PqqA peptide cyclase	Cytoplasmic	PA1989 (*pqqE*) (QC 99%; ID 77%)	Q88QV8
PP_0377	*pqqD-I*	PqqA binding protein (chaperone)	Cytoplasmic	PA1988 (*pqqD*) (QC 93%; ID 60%)	Q88QV7
PP_0378	*pqqC*	Pyrroloquinoline-quinone synthase	Cytoplasmic	PA1987 (*pqqC*) (QC 97%; ID 84%)	Q88QV6
PP_0379	*pqqB*	Hydrolase	Cytoplasmic	PA1986 (*pqqB*) (QC 100%; ID 73%)	Q88QV5
PP_0380	*pqqA*	Peptide precursor	Cytoplasmic	PA1985 (*pqqA*) (QC 100%; ID 83%)	Q88QV4
PP_0381	*pqqF*	Protease	(Periplasmic)	PA1973 (*pqqF*) (QC 90%; ID 53%)	Q88QV3
Other relevant proteins outside of the cluster in *P. putida* KT2440					
PP_4362	–^*d*^	Histidine phosphotransferase	Cytoplasmic	PA3345 (QC 95%; ID 51%)	Q88EU1
PP_1635	*erdR* (*crbR*)	DNA-binding response regulator	Cytoplasmic	PA3604 (*erdR*) (QC 100%; ID 83%)	Q88MD8
PP_1695	*mxtR*	Hybrid histidine kinase	Cytoplasmic membrane	PA3271 (*crbS*) (QC 100%; ID 79%)	Q88M79
PP_4695	*cbrA*	Histidine kinase	Cytoplasmic membrane	PA4725 (*crbA*) (QC 100%; ID 82%)	Q88DX3
PP_4696	*cbrB*	σ^54^-dependent response regulator	Cytoplasmic	PA4726 (*cbrB*) (QC 99%; ID 84%)	Q88DX2

^*a*^ Predicted function based on UniProt and InterPro annotations, indicated in parentheses.

^*b*^ Predicted localization based on PSORTb (v.3.0.2) (34) and SignalP (35) analyses; proteins predicted in multiple compartments (excluding the cytoplasm) are classified as “non-cytoplasmic”; predictions are indicated in parentheses.

^*c*^ QC, query cover; ID, identity.

^*d*^ –, not applicable.

Interestingly, *agmR* expression varies under different physiological conditions. In *P. extremaustralis*, for instance, expression increases at low temperatures, possibly to promote energy conservation (44). In *P. putida* KT2440, AgmR has been associated with chloramphenicol resistance, although the use of ethanol as a solvent for the antibiotic may have contributed to this result (119). Disruption of *agmR* reduced growth on short-chain alcohols including ethanol, isoprenol, pentanol, and butanediols in *P. putida* KT2440 and *P. putida* U (5, 15). In turn, the activity of AgmR itself may be regulated by upstream components, for example, PP_2683 and YiaY. AgmR also appears to act upstream of the PedS2-PedR2 system (18, 19, 65, 114), as shown in Fig. 3a.

#### The REE-responsive regulators PedS2-PedR2

The histidine kinase PedS2 and the LuxR-type response regulator PedR2, also known as ExaD or EraS and ExaE or EraR, respectively, form a conserved TCS encoded within the *ped* cluster in multiple *Pseudomonas* species. This system controls the regulation of PQQ-dependent alcohol dehydrogenases based on the availability of REEs (18, 36, 65, 118). Under REE-rich conditions and in the presence of alcohols, PedS2-PedR2 represses expression of the Ca^2+^-dependent *pedE* gene and simultaneously induces the REE-dependent *pedH* (Fig. 2). In contrast, the expression of *pedE* is favored when REEs are absent (65). Although PedS2 is anchored to the cytoplasmic membrane, its HAMP sensory domain is located in the cytoplasm (Fig. 2). Activation of this signaling cascade requires intracellular transport of La^3+^ ions by the ABC transporter PedA1A2BC (27). The PedS2-PedR2 system is also found in pseudomonads that lack a functional *pedH* homolog, such as *P. aeruginosa* PAO1. This observation suggests regulatory functions beyond the known *pedH* activation (18, 19).

### The PQQ biosynthesis operon

Although the *pqq* biosynthetic operon is distant from the *ped* cluster in the genome of *P. putida* (Fig. 1 and Table 2), it is transcriptionally co-regulated. This regulation is likely mediated by the transcriptional regulator AgmR (117). In some *Pseudomonas* species, including *P. aeruginosa* PAO1, the *pqq* operon is located between *pedI* and *yiaY*. This gene cluster functions either as the primary biosynthetic locus or as a secondary copy (Fig. 1).

**Fig 4:**
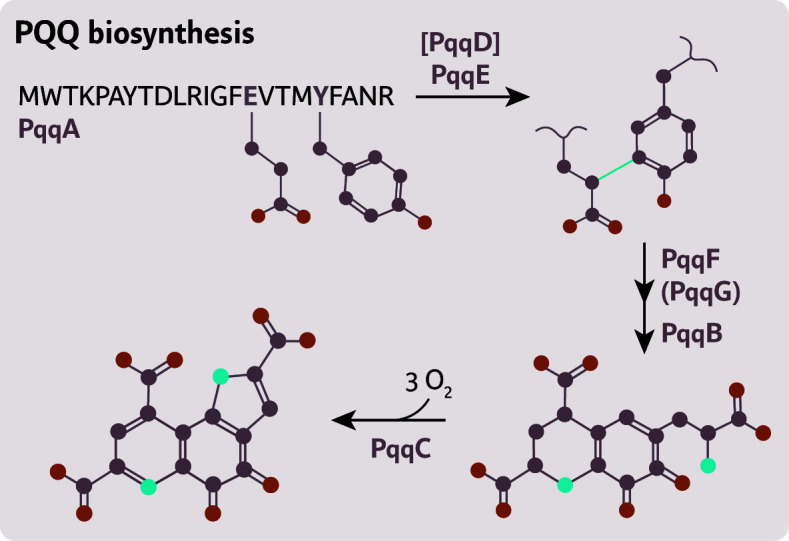
Outline of the PQQ biosynthesis pathway in *Pseudomonas* species catalyzed by the products of the *pqqFABCDE(G*) operon. PqqD functions as a chaperone that facilitates the interaction between PqqE and PqqA. PqqG can form heterodimers with PqqF, but it is not essential, as PqqF alone is sufficient to carry out the peptidase reaction.

PQQ cofactor biosynthesis begins with the peptide precursor PqqA, which contains conserved glutamate and tyrosine residues (Fig. 4). The radical *S*-adenosyl-L-methionine enzyme PqqE, together with the chaperone PqqD, catalyzes the initial cross-linking of these residues (17). The intermediate is then processed by the metallopeptidase PqqF. PqqC completes the final oxidative steps to produce mature, redox-active PQQ (Fig. 4). Studies on a PqqG homolog in *M. extorquens* showed that it can form a heterodimeric complex with PqqF to proteolytically process PqqA peptides. PqqF alone was also sufficient to degrade PqqA (120). Consistent with these findings, deletion of *pqqG* in *P. putida* did not impair growth on volatile alcohols. This indicates that PqqF can process PqqA independently (17).

The *pqq* operon is co-transcribed and becomes upregulated during aerobic growth on volatile organic compounds and glucose. This expression pattern is linked to the role of PQQ-dependent glucose dehydrogenases in glucose catabolism (13, 121). Functional studies show that disruption of any *pqq* gene—except *pqqG*—prevents PQQ biosynthesis and impairs alcohol metabolism. These findings highlight the essential role of the *pqq* genes (17). Several steps in the biosynthetic cascade proceed spontaneously but are strictly O_2_-dependent (13, 14), confining the activity of this pathway to aerobic environments or niches where external PQQ is available.

## CLUSTER REGULATION AT HIGHER HIERARCHICAL LEVELS

The *ped* gene cluster displays a complex regulatory structure defined by multilayered signal integration and transcriptional control. Although the complete regulatory framework remains fairly incomplete, experimental evidence gathered from experiments in different *Pseudomonas* species has advanced understanding of upstream elements and environmental signals that influence *ped* cluster activity. The regulatory complexity in *P. putida* exceeds that of *P. aeruginosa* due to the presence of a REE-dependent ADH. Despite this scenario, transcriptional output appears largely conserved, indicating the presence of similar regulatory systems and environmental sensing pathways (Fig. 3a).

In *P. aeruginosa* PAO1 (18, 19), the two-component system CrbRS has been proposed as an upstream transcriptional activator of *yiaY*, *PP_2683* (known as *ercAS*), and *agmR* homologs (Table 2). CrbRS also contributes to ethanol oxidation and acetate metabolism in other proteobacterial species, including *V. cholerae* and *Pseudomonas entomophila* (112, 122). Homologs of this system are present in *P. putida* KT2440. These include *erdR* (*PP_1635*) and *mxtR* (*PP_1695*), which encode a response regulator and a HHK with a sodium (Na^+^) or solute symporter-like domain, respectively (17, 123). Deletion of *erdR* or *mxtR* impaired growth on acetate but did not affect ethanol utilization (17, 123). These results align with earlier studies showing that ErdR and MxtR are required for acetate assimilation through activation of acetyl-coenzyme A synthetase in *P. putida* and *P. fluorescens* (124, 125). Whether this TCS serves as the initial regulator of the *ped* cluster in response to alcohols in *P. putida*, as in *P. aeruginosa*, is unresolved.

RB-*Tn*Seq experiments carried out in *P. putida* KT2440 have identified the CbrAB TCS (PP_4695–PP_4696) as a potential upstream regulator of the *ped* cluster (17). Disruption of *cbrA* or *cbrB* impaired growth on short-chain alcohols, for example, pentanol, but did not substantially affect growth on valerate (17). CbrAB is a global regulator of carbon catabolite repression, nitrogen-dependent control, and amino acid uptake in several *Pseudomonas* species (126, 127). Current evidence supports the hypothesis that CbrAB is the primary regulatory module initiating transcription of genes within the *ped* cluster in *P. putida* (Table 2 and Fig. 3a).

A secondary regulatory tier involves the HHK PP_2683, the transcriptional regulator AgmR, and the Fe^2+^-responsive alcohol dehydrogenase YiaY. All three components are required for full transcriptional activation of the *ped* cluster (Fig. 3). Although the exact signaling mechanism remains unresolved, AlphaFold3 structural models (110) predict direct interaction between the *N*-terminal domains of YiaY and PP_2683 (Fig. 3e). Hence, YiaY activity may stimulate the kinase function of PP_2683 (32, 111, 114). This mechanism resembles the NO-sensing NosP-NahK system in *P. aeruginosa* PAO1. In that system, homologs of PP_2663 and PedS1 regulate biofilm dispersal through HHK modulation by a dedicated sensing protein (102). It is unknown whether the YiaY-PP_2683 module transmits signals directly to AgmR or through an intermediary histidine phosphotransferase (Hpt; Fig. 3d). The conservation of these elements across *Pseudomonas* species suggests a shared regulatory framework (Fig. 3c).

Recent data suggest that PedS1 forms heterodimers with PP_2683, generating distinct HHK complexes that control substrate-specific transcriptional responses (111). For example, the *pedF* promoter activity decreased in a ∆*pedS1* strain exposed to ethanol, isopropanol, or 3-methyl-1-pentanol. However, the transcriptional activity increased in the presence of isoprenol or isopentanol (111), indicating differential regulation by homodimeric versus heterodimeric kinase assemblies (Fig. 3f). AlphaFold3 (110) predicts stable PedS1-PP_2683 dimerization (Fig. 3e), but the expression profiles of these components, particularly *pedS1*, remain to be clarified.

AgmR activation leads to transcriptional upregulation of most *ped* cluster genes and the distal *pqqFABCDEG* operon (Fig. 3a). Another regulatory axis involves the REE-dependent TCS PedS2-PedR2 (65), which governs the mutually exclusive expression of *pedE* (encoding a Ca^2+^-dependent enzyme) and *pedH* (a REE-dependent dehydrogenase) based on Ln^3+^ availability (Fig. 2 and 3a). In *P. aeruginosa* PAO1, the homologous EraSR system controls *exaA* expression, indicating functional conservation of Ln^3+^-responsive switches (18, 19).

Although several regulatory features remain unresolved, studies using gene deletions, promoter-reporter assays, and computational modeling are gradually clarifying the *ped* cluster control mechanisms. Proteins such as PedF and PedG, homologous to methylotrophic proteins, are increasingly suspected of participating in regulation. This possibility suggests a more integrated and evolutionarily conserved regulatory network (67, 96).

## GENE ORGANIZATION AND CLUSTER CONSERVATION

In Fig. 1, we have depicted the genomic conservation of the *ped* cluster. Through such taxonomic comparisons, the essentiality of the PedD and PedF regulators, together with at least one PQQ-ADH, becomes clear. Moreover, gene organization remains conserved, except for some *Stutzerimonas* or *Ectopseudomonas* species (Fig. 1b). Differences lie namely in the presence of a complete of a *pqq* biosynthesis operon inside the cluster (Fig. 1a and b) and a secondary PQQ-ADH (and its putative maturation machinery). Though PedH remains highly homologous to PedE and thus, it may have been a product of duplication, the presence of genes *pedG, PP_2677,* and *PP_2678* suggests that their origin could be from horizontal gene transfer from other methylotrophic or acetotrophic bacteria. Most importantly, the absence of the *ped* cluster in several strains of the groups of *P. putida* or *P. fluorescens* complicates the understanding of the cluster’s evolutionary trajectory (Fig. 1a). In fact, some strains of *P. resinovorans* or *P. chlororaphis* retained the presence of the cluster, whereas most of the isolated species do not possess a copy. Other *Pseudomonas* (sub-)groups do not seem to harbor an occurrence in their genome at all (e.g., *P. syringae*, *P. koreensis,* or *P. jessenii*). Such loss could be explained by the high overexpression of the cluster with substrate induction, which may pose a metabolic burden to the cell. On the other hand, the high activity of these dehydrogenases contributes to the increased formation of toxic aldehydes, which may not be further metabolized. And although *Pseudomonas* species are known for their increased tolerance to such compounds, cluster deletion may have been a protective mechanism towards these potential adverse effects. Nonetheless, further elucidation must be centered on this regard to draw significant conclusions.

## CONCLUDING REMARKS

PQQ-dependent ADHs, such as those encoded in the *ped* cluster of *Pseudomonas* species, illustrate how alcohol oxidation can be organized as a multi-component and tightly regulated system. Similar strategies operate in other ethanol-oxidizing bacteria, including acetic acid bacteria (128) and methylotrophs (70), where PQQ-dependent dehydrogenases support efficient oxidation of alcohols and aldehydes under aerobic conditions. Although these systems are more complex than single-gene NADH-dependent dehydrogenases, their organization into dedicated clusters enables high catalytic efficiency, regulatory inducibility, and integration with electron transport chains. This level of complexity reflects an evolutionary tradeoff in which increased genetic and regulatory investment supports metabolic flexibility, redox balancing, and adaptation to fluctuating environmental substrates.

Moreover, these enzymes display pronounced metabolic versatility and remain active under changing environmental conditions. Such resilience highlights their ecological relevance and potential for environmental and industrial biotechnological applications. Despite ongoing research, key aspects of the cluster’s function and regulatory mechanisms remain unresolved. The high conservation of this cluster across diverse *Pseudomonas* taxa suggests that further analysis in model species such as *P. putida* KT2440 and *P. aeruginosa* PAO1 will support identification of related systems in lesser-studied strains with broader or more specialized catabolic traits. A detailed mechanistic understanding of the regulation and coordination among cluster components will improve its applicability in synthetic methylotrophy, bioremediation, and related biotechnological applications (5, 32, 33). Together, the findings summarized in this review define a framework for future experimental studies that target both mechanistic dissection and applied exploitation of the *ped* cluster in pseudomonads. These directions include rational pathway engineering, regulatory rewiring, and integration of Ped-mediated alcohol oxidation into strategies for the synthetic assimilation of one-carbon substrates (129, 130), as well as extension to other bacterial species with biotechnological potential. As such, this perspective outlines avenues where the Ped system can be developed as a modular platform for applied microbiology.
